# Clustering and Source Association of Clinical and Nonclinical *Listeria monocytogenes* Isolates, New York, USA, 2000–2021^1^

**DOI:** 10.3201/eid3209.260289

**Published:** 2026-09

**Authors:** Hilal Samut, Damaris V. Mendez-Vallellanes, Hannah Hoyt, Samantha E. Wirth, Lisa Mingle, Brian D. Sauders, Gregory A. Deiulio, Alyssa W. Dickey, Maria L. Ishida, William J. Wolfgang, Kimberlee A. Musser, Martin Wiedmann, Renato H. Orsi

**Affiliations:** Cornell University, Ithaca, New York, USA (H. Samut, M. Wiedmann, R.H. Orsi); New York State Department of Health, Wadsworth Center, Albany, New York, USA (D.V. Mendez-Vallellanes, H. Hoyt, S.E. Wirth, L. Mingle, W.J. Wolfgang, K.A. Musser); New York State Department of Agriculture and Markets, Albany (B.D. Sauders, G.A. Deiulio, A.W. Dickey, M.L. Ishida)

**Keywords:** food safety, bacteria, Listeria monocytogenes, clonal complex, foodborne, surveillance, epidemiology, persistence, outbreak, dairy, meat, fish, produce, New York

## Abstract

We analyzed whole-genome sequencing data for 1,046 human clinical and 1,332 nonclinical *Listeria monocytogenes* isolates collected across New York, USA, during 2000–2021. Several hypervirulent clonal complexes (CCs) were significantly associated with clinical isolates, and several hypovirulent CCs were associated with nonclinical isolates. Specific CCs also showed association with specific food categories (e.g., processed meat); specific genetic markers (e.g., *inlA* premature stop codons) were also significantly associated with processed meat isolates. Analysis of clusters that contained food isolates, as well as subsequently identified clinical isolates, showed that time of isolation between food isolates and clinical isolates was significantly shorter for produce isolates than for isolates from meat, dairy, or fish. This finding suggests unique transmission pathways for produce, which might reflect short shelf life or limited *L. monocytogenes* persistence (e.g., in agricultural environments). This study highlights new opportunities for use of whole-genome sequencing to improve outbreak investigations and source attribution.

*Listeria monocytogenes* is a leading cause of foodborne illness–related deaths in the United States; an estimated 1,250 human illnesses and 172 deaths occurred annually during 2016–2019 (*1*). *L. monocytogenes* is frequently isolated from dairy, ready-to-eat meats, seafood, fruits, and vegetables, and it can persist in food processing environments (*2*), leading to recurrent contamination of finished products. Hence, robust surveillance is critical for protecting vulnerable populations and rapid detection of contamination sources.

Multilocus sequence typing (MLST) is widely used to classify *L. monocytogenes* into clonal complexes (CCs), and certain CCs are suggested as presenting higher and lower virulence (*3*). CC1, CC4, and CC6 are considered hypervirulent and are frequently associated with human listeriosis cases (*4*). Conversely, CC9 and CC121 are frequently isolated from food or food-processing environments and are considered hypovirulent CCs (*4*,*5*). Hypovirulent CCs often carry adaptations that promote environmental persistence (e.g., stress tolerance genes) but might have reduced ability to cause invasive disease (*6*). Characterizing the distribution of CCs across clinical and nonclinical isolates is key for identifying contamination reservoirs and understanding the public health relevance of isolates found in foods. Distinguishing hypervirulent strains from those mainly associated with the environment can help prioritize risk management and improve listeriosis prevention strategies (*7*). In this study, we analyzed 2,371 clinical and nonclinical *L. monocytogenes* isolates collected across the state of New York (NY), USA, during 2000–2021 to compare the CC distributions among clinical and nonclinical isolates, examine associations between CCs and food categories, and evaluate the time between food isolate collection and subsequent detection of a genetically-related clinical isolate using single-nucleotide polymorphism (SNP)–based thresholds. The Wadsworth Center and Cornell University Institutional Review Boards reviewed this study and determined it to be exempt from human research (submission no. 03-037 and IRB0150826).

## Materials and Methods

Detailed materials and methods are provided in the Appendix. In brief, we performed whole-genome sequencing (WGS) on clinical (n = 1,046) and nonclinical (n = 1,332) *L. monocytogenes* isolates collected across NY, as previously described (*8*). We deduplicated clinical isolates from the same patient or mother–infant pairs, resulting in 1,006 clinical isolates. Likewise, we deduplicated multiple nonclinical isolates from the same sampling event, resulting in 1,089 nonclinical isolates.

We used whole-genome SNP distances to assess the genetic relatedness among isolates, as previously described (*8*). We used genetic relatedness of clinical and nonclinical isolates to define 3 types of isolate grouping. When only clinical isolates were involved, we used the term clustered, and when clinical and nonclinical isolates were involved, we used the term linked. Hence, the term linked was used to describe genomic similarity between clinical and nonclinical isolates on the basis of SNP thresholds, rather than confirmed epidemiologic associations. Therefore, NY clinical isolates were considered clustered if clustered with >1 other NY clinical isolate by <20 SNPs (*9*–*11*), NY nonclinical isolates were considered cluster-linked if linked by <50 SNPs with >1 clustered NY clinical isolate (*12*), and NY clinical and nonclinical isolates were considered sporadic-linked if a NY clinical isolate was not clustered with any other NY clinical isolate by <20 SNPs but was linked with >1 NY nonclinical isolate by <50 SNPs. The terms clustered, cluster-linked, and sporadic-linked refer only to genetic relatedness on the basis of SNP thresholds, without interpretation of epidemiologic data. Isolates not meeting those criteria were considered unclustered NY clinical isolates and unlinked NY nonclinical isolates.

## Results

### CC Distributions among Clinical and Nonclinical Isolates

**Figure 1:**
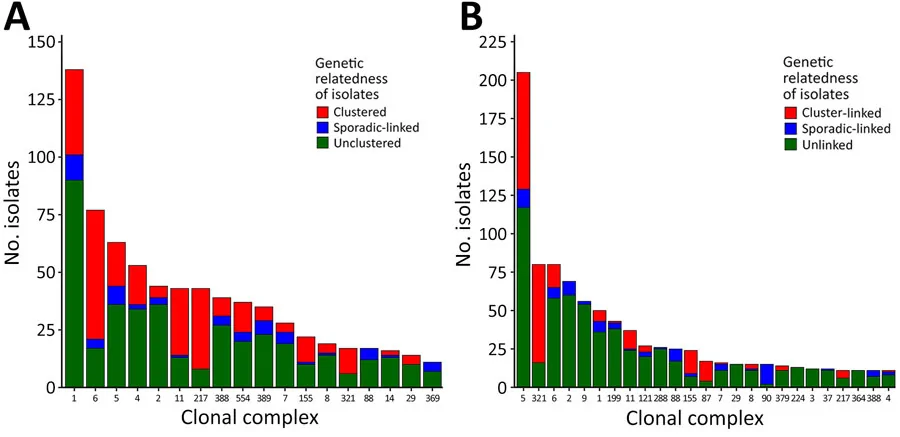
Distribution of *Listeria monocytogenes* CCs by grouping status in terms of genetical relatedness by single-nucleotide polymorphism (SNP) distances in study of clustering and source association of clinical and nonclinical *L. monocytogenes* isolates, New York, USA, 2000–2021. Distribution is shown for clinical (A) and nonclinical (B) isolates. Clinical isolates were considered clustered if they clustered with >1 other clinical isolate by <20 SNPs and nonclinical isolates were considered cluster-linked if they were linked with >1 clustered clinical isolate by <50 SNPs; both clinical and nonclinical isolates were considered sporadic-linked if the clinical isolate was not clustered with any other clinical isolate by <20 SNPs but linked with >1 nonclinical isolate by <50 SNPs. The terms clustered, cluster-linked, and sporadic-linked refer only to genetic relatedness based on SNP thresholds, without interpretation of epidemiologic data. Clinical isolates not meeting those criteria were considered unclustered, while nonclinical isolates were considered unlinked. CCs representing <1% of total isolates within each isolate type were grouped as other and excluded from this figure. CC, clonal complex.

**Figure 2:**
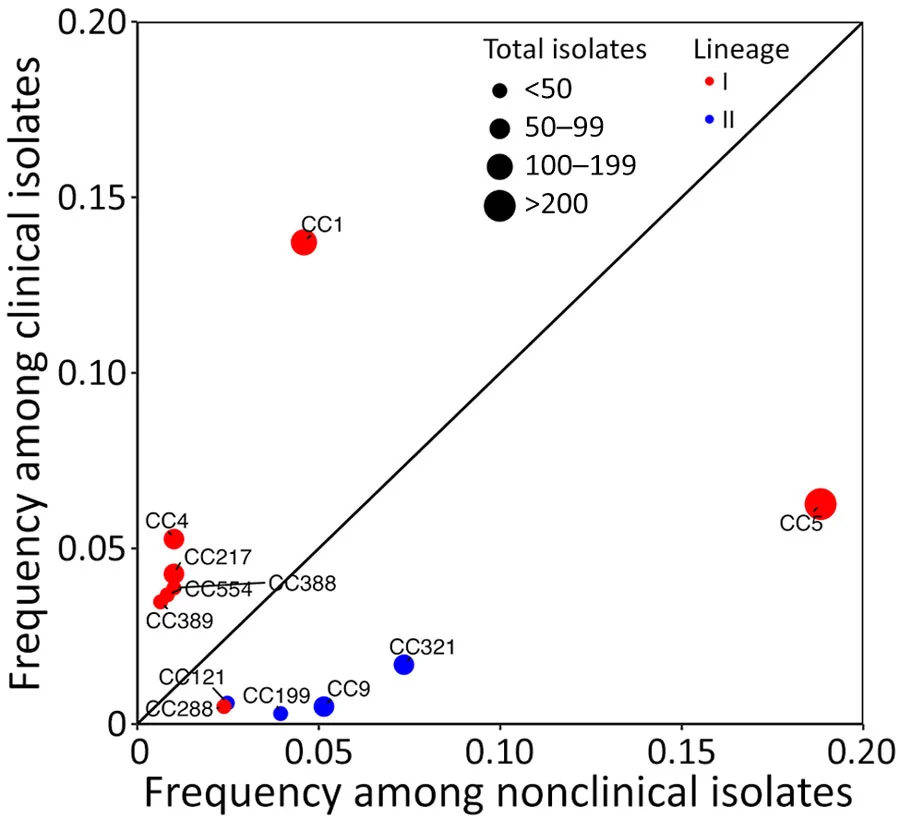
Comparison of the relative frequency of *Listeria monocytogenes* CCs among clinical and nonclinical isolates in study of clustering and source association of clinical and nonclinical *L. monocytogenes* isolates, New York, USA, 2000–2021. Each bubble represents CCs that were found to be significantly overrepresented among either clinical or nonclinical isolates (Table 1). Diagonal reference line indicates equal frequency in clinical and nonclinical isolates; CCs above the line were common in clinical isolates, whereas CCs below were common in nonclinical isolates. CC, clonal complex.

Among clinical isolates, CC1 (n = 138), CC6 (n = 77), CC5 (n = 63), and CC4 (n = 53) were the most prevalent CCs (Figure 1, panel A). Among nonclinical isolates, CC5 (n = 205), CC321 (n = 80), CC6 (n = 80), and CC2 (n = 69) were the most prevalent CCs (Figure 1, panel B). CC1, CC4, CC217, CC388, CC389, and CC554 were significantly overrepresented among clinical isolates, whereas CC5, CC9, CC121, CC199, CC288, and CC321 were significantly overrepresented among nonclinical isolates (Table 1; Figure 2).

**Table 1:** *Listeria monocytogenes* clonal complexes significantly associated with either clinical or nonclinical isolates in study of clustering and source association of clinical and nonclinical *Listeria monocytogenes* isolates, New York, USA, 2000–2021*

CC	No. clinical isolates	No. nonclinical isolates	OR (95% CI)†	FDR-adjusted p value‡
CCs overrepresented among clinical isolates				
CC1	138	50	3.30 (2.34–4.72)	<0.001
CC4	53	11	5.45 (2.79–11.63)	<0.001
CC217	43	11	4.37 (2.20–9.46)	<0.001
CC388	39	11	3.95 (1.97–8.60)	<0.001
CC389	35	7	5.57 (2.42–14.93)	<0.001
CC554	37	9	4.58 (2.16–10.85)	<0.001
CCs overrepresented among nonclinical isolates				
CC5	63	205	0.29 (0.21–0.39)	<0.001
CC9	5	56	0.09 (0.03–0.23)	<0.001
CC121	6	27	0.24 (0.08–0.59)	<0.001
CC199	3	43	0.07 (0.01–0.23)	<0.001
CC288	5	26	0.20 (0.06–0.54)	<0.001
CC321	17	80	0.22 (0.12–0.37)	<0.001

*Only CCs with an FDR-adjusted p value <0.05 are shown. CC, clonal complex; FDR, false discovery rate; OR, odds ratio. †ORs >1 indicate overrepresentation in clinical isolates, whereas ORs <1 indicate overrepresentation in nonclinical isolates. ‡Statistical significance was assessed using 2-sided Fisher exact test followed by FDR correction for multiple comparisons.

On the basis of their grouping patterns within NY isolates, 318 (31.6%) clinical isolates were clustered, 79 (7.9%) were sporadic-linked, and 609 (60.5%) were unclustered (Figure 1, panel A). Among nonclinical isolates, 236 (21.7%) were classified as cluster-linked, 107 (9.8%) were sporadic-linked, and 746 (68.5%) were unlinked (Figure 1, panel B). Most CC321 nonclinical isolates were cluster-linked, whereas CC6, CC2, CC9, and CC121 nonclinical isolates were predominantly unlinked (e.g., 59 out of 61 CC9 nonclinical isolates were unlinked).

### Association of CCs with Food Categories in NY

**Figure 3:**
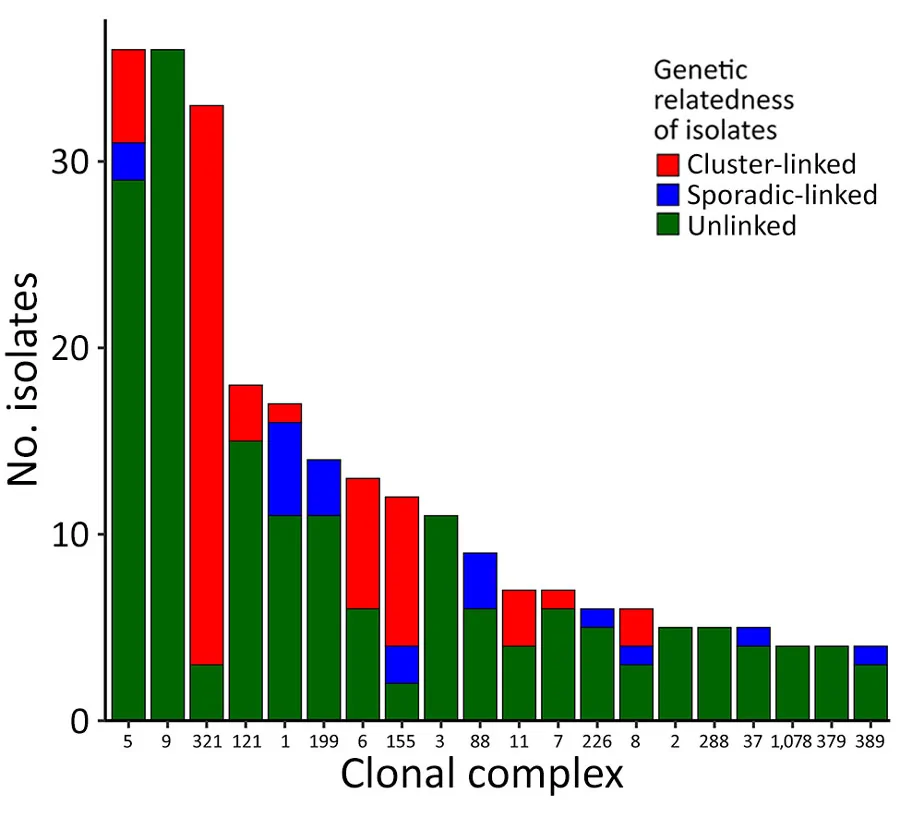
Distribution of *Listeria monocytogenes* clonal complexe (CCs) by grouping status in terms of genetical relatedness by single-nucleotide polymorphism (SNP) distances among food isolates in study of clustering and source association of clinical and nonclinical *L. monocytogenes* isolates, New York, USA, 2000–2021. Nonclinical isolates were considered cluster-linked if they were linked with >1 clustered clinical isolate by <50 SNPs, and nonclinical isolates were considered sporadic-linked if a clinical isolate was not clustered with any other clinical isolate by <20 SNPs but linked with >1 non-clinical isolate by <50 SNPs. Nonclinical isolates not meeting those criteria were considered unlinked. CCs representing <1% of total isolates within each isolate type were grouped as other and excluded from this figure.

The 313 food isolates (28.7% of nonclinical isolates) came from processed meat (n = 93), processed fish (n = 72), raw dairy (n = 47), raw fish (n = 22), processed dairy (n = 21), raw meat (n = 21), produce (n = 15), sandwiches (n = 9), salad (n = 8), pasta (n = 4), and nuts (n = 1). The most prevalent CCs among food isolates were CC5 (n = 36), CC9 (n = 36), and CC321 (n = 33) (Figure 3). Of the 313 food isolates, 69 were cluster-linked, 25 were sporadic-linked, and 219 were unlinked. Some CCs (e.g., CC5, CC9, CC121) included exclusively or predominantly unlinked food isolates. In contrast, most CC321 and CC155 food isolates were cluster-linked.

**Figure 4:**
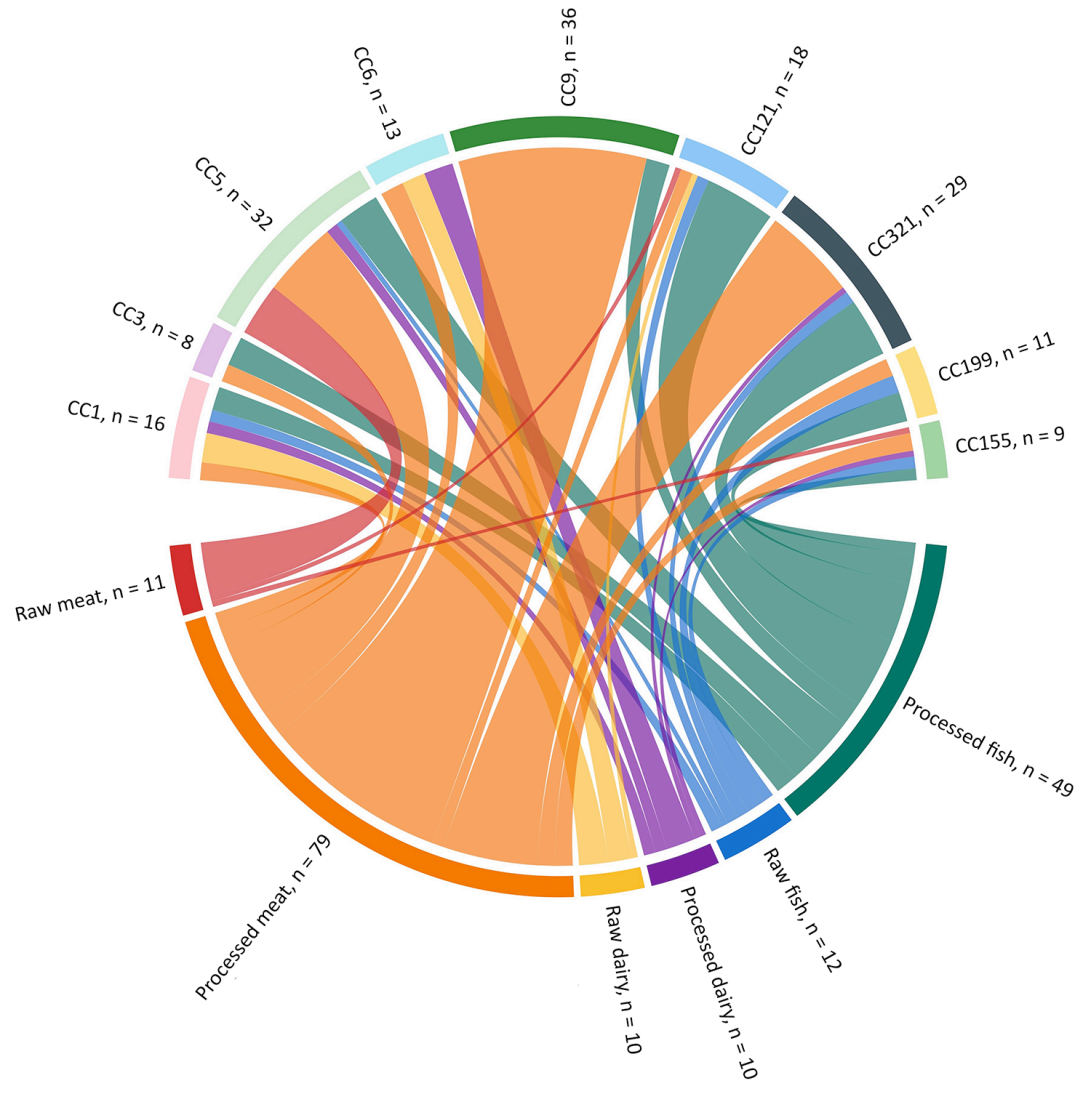
Sankey diagram showing the distribution of *Listeria monocytogenes* CCs across major food categories in study of clustering and source association of clinical and nonclinical *L. monocytogenes* isolates, New York, USA, 2000–2021. Only CCs with >10 food isolates are shown. Food categories with <20 isolates (e.g., produce, salad, pasta, nuts, and sandwiches) were excluded for clarity. The width of each flow is proportional to the number of isolates linked with a specific food category (bottom) and a CC (top). CC, clonal complex.

Some CCs were significantly associated with certain food categories (Table 2; Figure 4), including raw or processed forms of the same type (e.g., raw or processed dairy). For example, CC9 was significantly associated with processed meat, CC5 with raw meat, and CC121 with processed fish. Other CCs were not statistically associated with certain food categories after correction for multiple testing (false discovery rate–adjusted p value>0.05) but tended to be more commonly found in a specific category. For example, CC6 was ≈11 times more likely to be isolated from processed dairy and 2 times more likely to be isolated from raw dairy compared with all other food categories (Appendix Table 1).

**Table 2:** Associations between *Listeria monocytogenes* clonal complexes and food categories in study of clustering and source association of clinical and nonclinical *Listeria monocytogenes* isolates, New York, USA, 2000–2021*

CC†	Food category‡	OR (95% CI)§	FDR-adjusted p value¶
CC5, n = 36	Raw meat, n = 9	7.34 (2.89–18.60)	0.019
CC9, n = 36	Processed meat, n = 32	25.42 (9.12–70.91)	<0.001
CC121, n = 18	Processed fish, n = 12	7.49 (2.78–20.12)	0.015
CC369, n = 3	Produce, n = 3	167.16 (8.18–3414.14)	0.015
CC389, n = 4	Raw dairy, n = 4	55.14 (2.92–1042.22)	0.043
CC573, n = 3	Produce, n = 3	167.16 (8.18–3414.14)	0.015
CC1078, n = 4	Raw dairy, n = 4	55.14 (2.92–1042.22)	0.043

*Only CC-food category pairs with an FDR-adjusted p value <0.05 are shown (7 out of 660 CC-food category pairs). CC, clonal complex; FDR, false discovery rate; OR, odds ratio. †n value indicates number of food isolates with respective CC. ‡n value indicates number of food isolates with respective food category and with corresponding CC. §A continuity correction of 0.5 was applied to all cells to allow for OR calculation and ensure comparability across categories. ORs >1 indicate overrepresentation of the CC in the corresponding food category. ¶Associations were assessed using 2-sided Fisher exact test followed by FDR correction for multiple comparisons.

We also performed further comparison for CCs with >10 food isolates (Appendix Table 2) to assess whether those CCs were specifically associated with the processed or raw forms of those key food categories (i.e., meat, fish, dairy). CC9 was more frequently observed among processed meat isolates and CC121 was more frequently observed among processed fish isolates than in raw meat (odds ratio [OR] 15.74 [95% CI 0.90–276.54]) and raw fish (OR 1.40 [95% CI 0.31–6.40]) isolates; however, those associations were not statistically significant after correction for multiple testing. Conversely, CC5 isolates were significantly overrepresented among raw meat compared with processed meat isolates (OR 20 [95% CI 4.17–100]), even after false discovery rate correction.

### Virulence and Sanitizer-Tolerance Genes

We screened clinical and nonclinical isolates for virulence and sanitizer tolerance genes (Appendix Figures 1, 2). The *Listeria* pathogenicity island (LIPI) 1, *inlA*, and *inlB* were nearly ubiquitous across both clinical and nonclinical isolates (>98%), whereas LIPI-3 and LIPI-4 showed higher prevalence among clinical isolates (43.64% for LIPI-3 and 19.48% for LIPI-4). We detected *inlA* premature stop codons (PMSCs) in 32 (3.18%) clinical and 217 (19.92%) nonclinical isolates; frequency was high among nonclinical-associated CCs such as CC9 (91%), CC199 (93%), and CC321 (97.50%). Among clinical isolates with *inlA* PMSC, 17 (53.12%) carried PMSC type 3 (truncated to 699 aa vs. the full-length 800 aa) and only 2 (6.25%) carried PMSC type 4 (8 aa). Among nonclinical isolates with *inlA* PMSC, PMSCs type 4 (79 [36.40%]) and type 3 (78 [35.94%]) predominated. PMSC type 4 was rarer among clinical isolates (n = 3 [0.30% of clinical isolates]) than in nonclinical isolates (n = 79 [7.25% of nonclinical isolates]). The remaining PMSCs spanned 14 other PMSC types, resulting in InlA proteins of varying lengths (Table 3). Sanitizer tolerance genes (*bcrABC, Tn6188_qac,* or *LGI1_LM5578_1862*) were detected in 354 nonclinical isolates (32.51% of nonclinical isolates; OR 5.81 [95% CI 4.44–7.67]). We noted significant overrepresentation of *inlA* PMSCs among processed meat isolates (Table 4). In contrast, *inlA* PMSCs were rare or absent among dairy product isolates (0 of 47 raw dairy isolates; 1 of 21 processed dairy isolates) (Table 4). Sanitizer tolerance genes were significantly overrepresented among isolates from processed fish (OR 3.00 [95% CI 1.74–5.16]) and underrepresented among raw dairy isolates (OR 0.02 [95% CI 0–0.30]) (Appendix Table 3).

**Table 3:** Summary of detected *inlA* premature stop codons in study of clustering and source association of clinical and nonclinical *Listeria monocytogenes* isolates, New York, USA, 2000–2021*

CCs with respective *inlA* allele	*inlA* allele IDs with detected PMSCs†	PMSC type (length of truncated protein)‡	No. observed clinical and nonclinical isolates with respective *inlA* allele	Sources of nonclinical isolates with respective *inlA* allele
CC5	138	1 (605 aa)	6 clinical isolates and 18 nonclinical isolates	Raw meat, n = 6; processed meat, n = 4; sandwich, n = 1; salad, n = 1; retail, n = 6
CC9	43	8 (459 aa)	5 nonclinical isolates	Processed meat, n = 3; retail, n = 2
CC9	44	11 (684 aa)	1 nonclinical isolate	Processed fish, n = 1
CC9	47	12 (576 aa)	5 nonclinical isolates	Processed meat, n = 1; retail, n = 4
CC9	48	4 (8 aa)	2 clinical isolates and 39 nonclinical isolates	Processed meat, n = 26; processed fish, n = 1; retail, n = 10; farm, n = 2
CC9	69	19 (326 aa)	1 nonclinical isolate	Processed meat, n = 1
CC121	49	6 (491 aa)	3 clinical isolates and 17 nonclinical isolates	Processed fish, n = 12; processed meat, n = 1; processing environment, n = 4
CC121	438	5 (188 aa)	2 clinical isolates and 6 nonclinical isolates	Raw meat, n = 1; processed meat, n = 1; raw fish, n = 1; retail, n = 1
CC193	41	25 (25 aa)	1 nonclinical isolate	Processed fish, n = 1
CC199	28	4 (8 aa)	1 clinical isolate and 40 nonclinical isolates	Processed fish, n = 5; raw fish, n = 3; processed meat, n = 3; produce, n = 1; nuts, n = 1; retail, n = 26; processing environment, n = 1
CC224	125	2 (655 aa)	1 clinical isolate and 2 nonclinical isolates	Processed fish, n = 1; salad, n = 1
CC321	91	3 (699 aa)	17 clinical isolates and 78 nonclinical isolates	Processed meat, n = 16; processed fish, n = 10; sandwiches, n = 3; processed dairy, n = 1; raw fish, n = 1; salad, n = 1; processing environment, n = 20; retail, n = 26
CC635	439	7 (561 aa)	5 nonclinical isolates	Processing environment, n = 5
ST13	35	15 (76 aa)	1 nonclinical isolate	Processed meat, n = 1

*CC, clonal complex; ID, identification number; PMSC, premature stop codon. †These allele IDs refer to IDs listed at https://bigsdb.pasteur.fr. ‡The classification of *inlA* PMSC types is based on Maury et al. (*4*) and Van Stelten et al. (*13*).

**Table 4:** Association between food category and presence of *inlA* premature stop codon among 313 *Listeria monocytogenes* food isolates in study of clustering and source association of clinical and nonclinical *L. monocytogenes* isolates, New York, USA, 2000–2021*

Food category	OR (95% CI)†	FDR-adjusted p value
Raw dairy	0.01 (0.00–0.24)	<0.001
Processed dairy	0.12 (0.02–0.65)	0.006
Produce	0.18 (0.03–0.98)	0.064
Pasta	0.20 (0.01–3.76)	0.474
Raw fish	0.56 (0.20–1.49)	0.462
Raw meat	0.95 (0.38–2.36)	1.000
Salad	1.18 (0.30–4.58)	1.000
Sandwich	1.52 (0.43–5.42)	0.886
Processed fish	1.55 (0.91–2.65)	0.270
Processed meat	4.93 (2.94–8.27)	<0.001
Nuts	5.58 (0.22–138.02)	0.483

*FDR, false discovery rate; OR, odds ratio. †OR >1 indicates overrepresentation of *inlA* premature stop codon presence in the corresponding food category. Associations were assessed using 2-sided Fisher exact test followed by FDR correction for multiple comparisons. A continuity correction of 0.5 was applied to all cells to allow for OR calculation and ensure comparability across categories.

### Influence of Food Category on Time between Isolate Collection and Detection of Genetically Related Clinical Isolate

We used a Cox proportional hazards model to evaluate the time between food isolate collection and subsequent detection of a genetically related clinical isolate (<50 SNPs) for food categories with >10 isolates (Appendix). Overall, we identified 205 food–clinical isolate pairs; 43 distinct food isolates (13 processed meat, 12 processed fish, 6 raw dairy, 4 processed dairy, 4 produce, 3 salad, and 1 raw meat) were linked with 107 distinct clinical isolates, 1 of which was linked with multiple food sources (processed meat and processed fish) at the same SNP distance. Processed meat (n = 126 pairs) and processed fish (n = 23 pairs) isolates had the highest number of links to clinical isolates (Table 5). The 205 food–clinical pairs were observed in 24 distinct SNP clusters and represented 17 distinct CCs (Table 5). Among the 43 food isolates, 10 of 13 processed meat isolates and 9 of 12 processed fish isolates had *inlA* PMSC. Out of 205 food–clinical isolate pairs, only 48 (23%) pairs with 16 distinct clinical isolates were involved in 4 different formal outbreak investigations. Among those, only 2 outbreak investigations had implicated food sources (processed meat in both outbreaks) for 13 clinical isolates; for those 2 outbreaks, the implicated food sources were consistent with the food category (i.e., processed meat) identified in our analysis.

**Table 5:** Characteristics of food-clinical *Listeria monocytogenes* isolate pairs used in the analysis of time between food isolate collection and subsequent detection of a genetically related clinical isolate by SNP cluster in study of clustering and source association of clinical and nonclinical *L. monocytogenes* isolates, New York, USA, 2000–2021*

CC	SNP cluster	Food category	No. food–clinical isolate pairs†	No. distinct food isolates	No. distinct clinical isolates linked with food isolates by<50 SNPs	*inlA* PMSC types in food and clinical isolates‡
CC1	PDS000003335§	Processed dairy	1	1	1	None
	PDS000025547	Processed meat	3	1	3	None
	PDS000082531	Processed dairy	1	1	1	None
	PDS000091530	Raw dairy	1	1	1	None
CC5	PDS000024625	Processed dairy	1	1	1	None
	PDS000024647	Salad	7	1	7	None
CC6	PDS000025014	Processed dairy	5	1	5	None
	PDS000025078§	Processed meat	23	1	23	None
CC7	PDS000003277	Processed meat	3	1	3	None
CC8	PDS000025311	Raw dairy	2	1	2	None
		Processed fish	1	1	1	None
CC88	PDS000003204	Processed fish	3	1	3	None
CC121	PDS000024645	Processed fish	2	2	1	Type 6
CC155	PDS000024385	Salad¶	1	1	1	None
	PDS000024989§	Raw meat¶	13	1	13	None
CC199	PDS000083553#	Processed fish	2	2	1	Type 4
		Processed meat	1	1	1	Type 4
CC204	PDS000003073	Salad¶	2	1	2	None
CC226	PDS000025453	Raw dairy	1	1	1	None
CC321	PDS000000366§	Processed fish	7	5	3	Type 3
		Processed meat¶	96	9	12	Type 3
CC369	PDS000090530	Produce	1	1	1	None
CC388	PDS000043055	Raw dairy	1	1	1	None
CC389	PDS000024852	Raw dairy	1	1	1	None
CC554	PDS000003170	Processed fish	8	1	8	None
	PDS000003173	Raw dairy	6	1	6	None
CC573	PDS000000308	Produce	12	3	5	None

*CC, clonal complex; PMSC, premature stop codon; SNP, single-nucleotide polymorphism. †Not all food–clinical isolate pairs met the <50 SNP threshold and, therefore, the number of food–clinical isolate pairs is not necessarily equal the number of food isolates times the number of clinical isolates in each SNP cluster. ‡When detected, *inlA* PMSC was present in all food and clinical isolates used in this analysis within the corresponding SNP cluster. When *inlA* PMSC was not detected, it was indicated as none. §Indicates SNP clusters that had formal outbreak investigations. In our analysis, PDS000003335 had 1 clinical isolate that was linked with 1 processed dairy isolate (by 2 SNPs); PDS000025078 had 9 clinical isolates that were linked with 1 processed meat isolate (3–9 SNPs); PDS000024989 had 2 clinical isolates that were linked with 1 raw meat isolate (0–2 SNPs), and PDS000000366 had 4r clinical isolates that were linked with 9 processed meat isolates (2–50 SNPs). Among those, 2 outbreak investigations (for the isolates in PDS000025078 and PDS000000366) had processed meat as the implicated food source, whereas the remaining investigations did not have a confirmed source. ¶These SNP clusters had other food categories matching to the clinical isolates by <50 SNPs. For clinical isolates associated with multiple food categories, only the clinical–food isolate pairs with the lowest pairwise SNP distance were retained. #For this SNP cluster, 2 food isolates from different food categories had the same minimum SNP distance to the same clinical isolate, and both food-clinical isolate pairs were retained.

**Figure 5:**
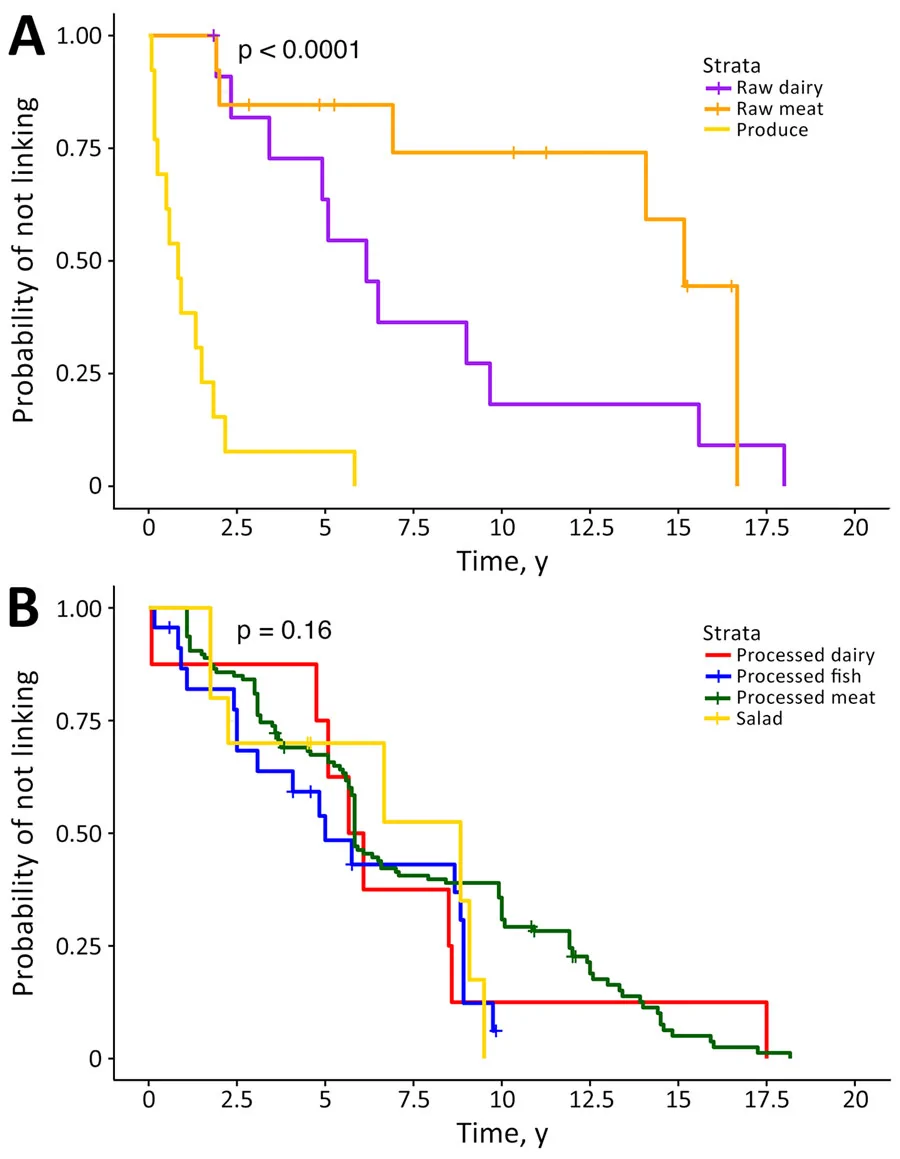
Kaplan-Meier survival curves for the time between food isolate collection and subsequent detection of a genetically related clinical *Listeria monocytogenes* isolate (single-nucleotide polymorphisms <50) by food category in study of clustering and source association of clinical and nonclinical *L. monocytogenes* isolates, New York, USA, 2000–2021. p<0.0001 indicates a significant difference in time between food isolate collection and subsequent detection of genetically related clinical isolate by single-nucleotide polymorphism cluster across food categories. A) Raw food categories (produce, raw meat and raw dairy); B) processed food categories (processed dairy, processed fish, processed meat, and salad). Salad refers to ready-to-eat mixed products that might involve additional handling steps and potential contamination during preparation or in retail establishments.

Food category, but not the presence of *inlA* PMSC, was a significant predictor of the time between food isolate collection and subsequent detection of a genetically related clinical isolate. In particular, produce isolates exhibited the shortest time for subsequent detection of a genetically related clinical isolate. For example, the hazard ratio for produce compared with processed dairy was 9.72 (95% CI 2.46–38.39), indicating a shorter time between produce isolate collection and subsequent detection of a genetically related clinical isolate than for processed dairy isolates (Appendix Table 4). Survival curves revealed that 75% of produce isolates were detected as genetically related with subsequently detected clinical isolates within 2.2 years; in contrast, some raw meat isolate pairs took up to 17 years to be genetically linked with a clinical isolate (Figure 5, panel A).

## Discussion

Our analysis of *L. monocytogenes* isolates in NY during 2000–2021 reveals clear differences in CC distributions between clinical and nonclinical isolates. Six CCs were significantly overrepresented among clinical isolates. Of those, 2 CCs (CC1 and CC4) have previously been identified as hypervirulent CCs on the basis of experimental evidence showing that they exhibit enhanced gut colonization, intestinal invasion, and infection of internal organs in a humanized mouse model (*4*). CC1, a well-established hypervirulent CC associated with invasive listeriosis (*14*,*15*), was the most frequently isolated CC among clinical isolates in our study, similar to a previous analysis of NY clinical isolates from 2000–2020 (*16*). The other CCs significantly overrepresented among clinical isolates in our study (i.e., CC217, CC388, CC389, and CC554) have not been phenotypically classified in terms of their virulence potential but might represent candidates for additional hypervirulent clones, as supported by studies suggesting that the virulence of *L. monocytogenes* CCs is positively associated with their epidemiologic association with human clinical cases (*3*). CC6 was the second most frequently isolated CC among clinical isolates but was not among the CCs significantly overrepresented among clinical isolates. That CC has previously been classified as hypervirulent and has been implicated in multiple large listeriosis outbreaks, including outbreaks linked to turkey deli meats, hot dogs, and French-style cheese (*17*). Previous studies in France and the Netherlands also identified CC1, CC2, CC4, and CC6 as frequently isolated CCs among clinical isolates (*3*,*18*). Overall, our data support that certain CCs are more likely to cause human disease.

We also identified 6 CCs (CC5, CC9, CC121, CC199, CC288, and CC321) that were significantly overrepresented among nonclinical isolates. CC9, CC121, CC199, and CC321 were the CCs with the highest proportion of isolates carrying *inlA* PMSC, a frequent hallmark of hypovirulent *L. monocytogenes* strains (*13*,*19*,*20*). CC9 and CC121 have previously been described as hypovirulent and environmentally adapted (*4*); those CCs were also reported to show increased tolerance to benzalkonium chloride, a commonly used sanitizer (*4*). Two CCs (CC5 and CC288) were overrepresented among nonclinical isolates but exhibited low frequencies of *inlA* PMSCs. Although CC5 was overrepresented among nonclinical isolates, it was the third most common CC among clinical isolates, suggesting that CC5 isolates are unlikely to be hypovirulent. The observed frequency of CCs among nonclinical isolates differs from observations in Europe. For example, CC121 was the most isolated CC among food and food processing environment–related isolates in France and Norway (*3*,*22*), as was CC9 in the Netherlands (*18*); CC121 and CC9 were the ninth and fifth most common CCs among nonclinical isolates characterized in this study. In contrast, CC5 was the most common CC among nonclinical isolates, but that CC was infrequently found among clinical and nonclinical isolates in France and the Netherlands (*3*,*18*). That discrepancy suggests a geographic difference in the distribution of this CC, which could be driven by possible differences in production systems, food preferences, or regional dynamics in *L. monocytogenes* transmission between NY and those countries in Europe. Despite likely regional differences in prevalence of different CCs, our data further support frequent global occurrence of certain clinically associated CCs (i.e., CC1 and CC4), whereas the occurrence of nonclinically associated CCs seems to show larger regional differences.

Overall, we found 7 statistically significant associations between CCs and food categories. For example, CC9 was significantly overrepresented among processed meat isolates and CC121 was significantly overrepresented among processed fish isolates. Those findings are consistent with previous reports indicating frequent recovery of those CCs from ready-to-eat meat and fish products and infrequent occurrence in dairy products (*4*,*22*–*24*). In addition, CC5 showed overrepresentation among raw meat isolates compared with processed meat isolates. That finding might suggest that different CCs could be introduced at distinct points along the food production chain; some CCs might be more closely linked to primary production or raw materials and others could be more likely to persist in food processing facilities. For the other 5 CCs associated with specific food categories, associations were based on smaller numbers (<10 isolates for the associated food category) and represented associations that had not been previously reported. Thus, those associations are possibly driven by specific sources (rather than general associations with a product type). Overall, our findings support that some CCs might be associated with certain food categories or with raw versus processed foods in a given category, which highlights opportunities to use WGS data to improve outbreak investigations and enable more rapid identification of a likely food source. However, although some CCs showed overrepresentation within specific food categories (e.g., CC9 with processed meat), others (e.g., CC321) were observed across multiple food sources with no overrepresentation in specific food categories. That finding suggests that certain CCs might not be specific to a single reservoir, and their presence should be interpreted cautiously in the context of source attribution.

Overall, *inlA* PMSCs were significantly overrepresented among isolates from processed meat and overrepresented, but they were not significantly overrepresented among processed fish isolates. Although our findings are consistent with previous studies that have reported frequent occurrence of *inlA* PMSC in isolates from processing plant environments and ready-to-eat foods (*13*,*19*,*21*,*25*), our analysis further highlights significant differences between processed and raw food isolates in the frequency of *inlA* PMSCs. Of note, a previous study in France also reported that hypovirulent CCs were associated with meat products in general (without clear indications on raw vs. processed sources) and proposed that hypovirulent CCs might be adapted to the processing environment, whereas hypervirulent isolates might be adapted to hosts (*4*). In contrast, we found that intact *inlA* were overrepresented among dairy isolates, which is also consistent with previous studies, such as a study in France that reported hypervirulent CCs to be strongly associated with dairy products (*13*). Finally, we found that intact *inlA* was borderline overrepresented among produce isolates (14 of 15 produce isolates showed intact *inlA*). That pattern is consistent with produce- and mushroom-focused surveys in the United States (*5*,*26*), United Kingdom (*27*), and China (*28*), which consistently reported low frequencies of *inlA* PMSCs in produce-associated isolates (*5*). In addition, we also found that sanitizer tolerance genes were significantly overrepresented in processed fish isolates and underrepresented in raw dairy isolates, consistent with a previous study that sanitizer tolerance genes were frequent among processed fish isolates (*5*). Overall, our findings suggest that food categories might differ in their likelihood of carrying virulence-attenuated *L. monocytogenes* and indicate that using WGS data to identify genetic markers (e.g., *inlA* PMSCs, sanitizer tolerance genes) could help with source attribution of raw versus finished products for some specific food categories.

We hypothesized that food category and presence of *inlA* PMSC were associated with the time between a food isolate collection and subsequent detection of a genetically related clinical isolate. Of note, we found that food category, but not the presence of *inlA* PMSC, was a significant predictor of that time; differences in time interval were observed across food categories. Specifically, isolates from produce had a shorter time for subsequent detection of a genetically related clinical isolate than did isolates from all other food categories.

Several factors likely contribute to the early detection of clinical isolates that are genetically related to produce isolates. Fresh produce typically has a shorter shelf life than other ready-to-eat products (e.g., cheese), possibly creating a narrow window in which contamination can directly lead to human exposure and illness. Contaminated produce typically would be consumed <20 days after processing, whereas cheese could be consumed >6 months after processing. However, the observed difference is unlikely to be explained solely by the short shelf life of fresh produce, because the time scale observed extends well beyond typical produce shelf life. Differences in consumer storage practices across different food categories (e.g., persons would more likely freeze raw meat than fresh produce) might extend the exposure window for certain food categories. In addition, *L. monocytogenes* contamination might be more frequently driven by repeated reintroduction rather than long-term persistence in packing and fresh-cut facilities (*29*–*31*). Persistent *L. monocytogenes* strains have also been detected less frequently in produce packinghouses (*31*–*33*) than in other food-associated facilities, although persistence over multiple seasons in some produce packing houses has been reported (*34*). Further research is needed to elucidate the persistence of *L. monocytogenes* in produce environments (*29*), but our findings suggest somewhat unique *L. monocytogenes* transmission pathways for produce. Of note, our data also showed that the time between the first food isolate and last subsequent detection of genetically linked clinical isolate is shorter for produce than for other food categories. That finding suggests that produce-linked clusters and outbreaks might often cover relatively short time frames, although long-term multistate outbreaks linked to produce have been reported (*35*). Even extremely small outbreaks putatively linked to produce might need to be further prioritized for outbreak investigations.

The first limitation of our study is that our dataset only included isolates obtained in NY during 2000–2021. Therefore, the proportion of unclustered and unlinked isolates might be overestimated because some clinical and nonclinical isolates could be clustered or linked with isolates from outside NY. Second, the SNP thresholds used to define clustering and linkage (i.e., <20 SNPs for clinical–clinical isolates and <50 SNPs for clinical–nonclinical isolates) are supported by previous studies as useful initial cut-offs for screening of potential clusters and sources but might need adjustment based on available epidemiologic data, particularly during outbreak investigations. Consequently, genetic relatedness inferred using those thresholds should be interpreted with caution. Third, genetic similarity alone does not establish epidemiologic linkage. Isolates with low SNP distances might reflect persistence in food production environments or widespread dissemination rather than direct transmission events. This study did not incorporate epidemiologic data (e.g., exposure histories), which limits the ability to confirm specific food sources for clinical cases. Similarly, a limitation of the cluster analysis is that some observed intervals between food isolate collection and subsequent detection of a genetically related clinical isolate were long. Those long intervals reduce confidence in interpreting the food as a continuous source of human exposure. Instead, such associations might reflect repeated reintroduction or cross-contamination across different reservoirs or food commodities. That interpretation is further supported by the observation that some SNP clusters included isolates from multiple food categories (Table 5). Therefore, the time intervals should be interpreted as reflecting temporal patterns of genomic relatedness rather than direct evidence of long-term source attribution to a single reservoir or commodity. Fourth, the dataset might be subject to biases, including uneven representation across food categories and time periods. In particular, the dataset includes isolates collected through targeted surveillance and research activities, which might result in overrepresentation of certain food categories or production environments. In addition, although thought to be rare, underdiagnosis or underreporting of listeriosis cases might lead to incomplete capture of clinical isolates. Finally, this study relied solely on SNP-based analyses. Incorporating approaches such as core-genome/whole-genome MLST could further strengthen the robustness of the findings, which highlights the need for more comprehensive integration of clinical and nonclinical isolates into unified databases to enable consistent genomic analyses, clustering, and outbreak investigations. In the future, advances in artificial intelligence and machine learning might further enhance the interpretation of surveillance datasets by identifying potential source attribution that are not easily analyzed through conventional approaches.

Appendix Additional information about clustering and source association of clinical and nonclinical *L. monocytogenes* isolates, New York, USA, 2000–2021.
