# Supplementary material for: Clustering and Source Association of Clinical and Nonclinical Listeria monocytogenes Isolates, New York, USA, 2000–2021
Source: Emerg Infect Dis. 2026 Sep;32(9):1430–40. doi: 10.3201/eid3209.260289 (PMC13555718; doi:10.3201/eid3209.260289)
Supplement: Supplementary file 1 [file 26-0289-Techapp-s1.pdf]

Article DOI: https://doi.org/10.3201/eid3209.260289

EID cannot ensure accessibility for supplementary materials supplied by authors. Readers who have difficulty accessing supplementary content should contact the authors for assistance.

Clustering and Source Association of Clinical and Nonclinical *Listeria monocytogenes* Isolates, New York, USA, 2000–2021

Appendix

Materials and Methods

1. Whole-genome sequencing of *Listeria monocytogenes* isolates

Whole-genome sequencing (WGS) of human clinical and nonclinical *L. monocytogenes* isolates was performed as explained in Samut et al. (*1*). Briefly, nonclinical isolates were obtained from food products collected and tested through routine sampling by the New York State Department of Agriculture and Markets (NYSAGM) Divisions of Food Safety and Inspection, Milk Control and Dairy Services, and Food Laboratory, and from prior studies completed in Cornell University Food Safety Laboratory (*2*,*3*). The isolates were sequenced using Illumina MiSeq platforms with Nextera XT or DNA Prep library kits. Clinical isolates obtained by New York State Department of Health (NYSDOH) and New York City Department of Health and Mental Hygiene (NYCDOHMH) were sequenced using the Illumina NextSeq 500 platform with quarter-scale DNA Prep library preparation, following the protocol by Dickinson et al. (*4*). WGS data for clinical isolates were submitted to PulseNet and National Center for Biotechnology Information (NCBI), where isolates were assigned to SNP clusters using a reference whole-genome multilocus sequence type (wgMLST) scheme and 25-allele cutoff, as detailed by Stevens et al. (*5*). Nonclinical isolates obtained by Cornell University Food Safety Laboratory included in this study (n = 507) are not part of PulseNet database but part of NCBI. From NCBI, the data was added to the Pathogen Detection (PD) browser. Pairwise SNP

distances were obtained from the NCBI-PD database. The publicly available WGS data were assigned the following BioProject accession numbers: PRJNA212117, PRJNA215355, PRJNA302599, PRJNA351287, PRJNA483181, PRJNA514286, PRJNA664209, PRJNA245909, PRJNA395587, PRJNA514285, PRJNA561882, PRJNA512470, PRJNA514288.

2. Isolate's metadata and data clean-up

The metadata and genetic relatedness data for clinical isolates and the deduplication of mother-child pairs and isolates from the same patient were detailed in Samut et al. (*1*), resulting in a final set of 1,006 clinical isolates used in this study. Briefly, pairwise SNP distances and associated metadata were downloaded from the NCBI PD database (accessed on October 4, 2023). Additional metadata, including exact collection dates, county-level location, source description, and outbreak or cluster codes, were obtained from NYSDOH, NYSAGM, and Food Microbe Tracker.

Nonclinical isolates included historical isolates obtained from food samples, food processing environments, retail establishments, animals and animal feces, and urban and natural environments. When nonclinical *L. monocytogenes* isolates was detected in more than one site (e.g., drains, sink, floor) in the same food processing facility or retail establishments during routine sampling (i.e., the *L. monocytogenes* isolates in our metadata were collected at the same exact sampling date and the same county) and the collected isolates were part of the same NCBI-assigned SNP cluster, only one instance of the isolates was kept in the dataset to prevent overrepresentation of multiple occurrences from the same facility or retailer as part of one sampling procedure. As a result, 243 nonclinical isolates represented more than once in the same facility or retail establishments in the same sampling event were excluded from the study by keeping only one isolate per instance. This resulted in a total of 1,089 nonclinical isolates including 313 food samples, 470 environmental samples from retail establishments, 89 food processing or packing facilities, 154 farms (e.g., water, feed, soil, pasture, water through), 52 domestic and wild animal sources (e.g., animal feces, clinical animal samples), and 11 natural and urban environments. The 1,089 de-duplicated nonclinical isolates were collected across 54 of the 62 NY counties (none from Warren, Schoharie, Montgomery, Hamilton, Fulton, Franklin, Clinton, and Cattaraugus counties). Kings county (n = 157) had the highest number of

nonclinical isolates, followed by Tompkins (n = 84), and Bronx (n = 68). County-level geolocation for 185 nonclinical isolates was unavailable.

Food samples were categorized based on the Interagency Food Safety Analytics Collaboration (IFSAC) food categorization scheme (*6*). To provide greater resolution, we further distinguished between raw and processed versions. Accordingly, we categorized food samples into processed dairy, raw dairy, processed fish, raw fish, processed meat, raw meat and produce categories. In addition to these, we also included several food categories that were not classified under the IFSAC grouping: sandwiches, salad, nuts, and pasta.

3. Multi locus sequence typing and clonal complex identification

For MLST sequence typing, CC and lineage identification of both clinical and nonclinical isolates, the assemblies were downloaded from NCBI PD (https://www.ncbi.nlm.nih.gov/pathogens/). For three isolates where assemblies were not available, the raw reads were retrieved from NCBI and assembled de novo using SKESA with default parameters (*7*). All *L. monocytogenes* assemblies were compared against the Institut Pasteur BIGSdb-Lm database (https://bigsdb.pasteur.fr/listeria/) for 7-gene MLST schemes (i.e., *abcZ, bglA, cat, dapE, dat, ldh, and lhkA*). Novel alleles (n = 4) and novel sequence type profiles (n = 5) were submitted to BIGSdb-Lm for allelic profile assignments. CC and lineage designations were retrieved for all isolates through BIGSdb-Lm (*8*). For the isolates containing novel alleles, the lineage identification was done using *sigB* allele database (*9*).

4. Identification of clustering between nonclinical and clinical isolates

Whole-genome SNP distances were used to assess the genetic relatedness among isolates as previously described (*1*). For clustering nonclinical isolates with clinical isolates, nonclinical isolates differing by ≤50 SNPs from clinical isolates in a SNP cluster were clustered using a single-linkage clustering approach. A loose threshold of ≤50 SNPs was used between clinical and nonclinical isolates for initial screening purposes, consistent with previous studies that used similar criteria to identify strains potentially persisting within the same facility (*10*). For the clustering of clinical isolates, a threshold of ≤20 SNPs was selected based on the maximum pairwise SNP distances typically observed in foodborne outbreak investigations (*11–13*). Genetical relatedness of clinical and nonclinical isolates were used to define three types of isolate grouping. When only clinical isolates are involved, the term “clustered” was used and

when clinical and nonclinical isolates were involved, the term “linked” was used; hence, here the term “linked” was used to describe genomic similarity between clinical and nonclinical isolates based on SNP thresholds, rather than confirmed epidemiologic associations. Therefore, (i) NY clinical isolates were considered **“clustered”** if clustered with ≥1 other NY clinical isolate by ≤20 SNPs (*11–13*), (ii) NY nonclinical isolates were considered **“cluster-linked”** if linked by ≤50 SNPs with ≥1 clustered clinical isolate (*10*), and (iii) NY clinical and nonclinical isolates were considered **“sporadic-linked”** if a NY clinical isolate was not clustered with any other NY clinical isolate by ≤20 SNPs, but was linked with ≥1 NY nonclinical isolate by ≤50 SNPs. The terms “clustered,” “cluster-linked,” and “sporadic-linked” here refer only to genetic relatedness based on SNP thresholds, without interpretation of epidemiologic data. Isolates not meeting these criteria were considered **“unclustered”** NY clinical isolates and **“unlinked”** NY nonclinical isolates.

5. Screening for virulence and tolerance genes and identifying premature stop codons (PMSCs)

The virulence and tolerance-associated genes were screened for presence across all isolate assemblies including virulence genes (*actA, hly, mpl, prfA, plcA, plcB, inlA, inlB,* LIPI-3 genes [*llsABDGHXYP*], LIPI-4 genes [*LM9005581_70009–70014*]), tolerance genes (*bcrABC, qacA, Tn6188_qac, cadA, cadC, LGI1_LM5578_1862*) and stress response genes SSI-1 (*lmo0444-lmo0448*), SSI-2 (*lin0464, lin0465*), and *comK.* Gene presence was determined using allele databases per gene from BIGSdb-Lm. A gene was considered present if the BLAST hit had ≥99% nucleotide identity and ≥90% query coverage; otherwise, it was considered absent. To detect PMSCs in *inlA*, nucleotide sequences aligned to minus strand were first reverse-complemented using Seqtk (https://github.com/lh3/seqtk). The sequences were then translated using SeqKit (*15*).

6. Survival model: Analysis of time between a food isolate collection and subsequent detection of genetically-related clinical isolates

To investigate whether food source influences the time between *L. monocytogenes* food isolate collection and subsequently detected genetically-related clinical isolates, a survival analysis was conducted. The outcome of interest was the time (in years) from food isolate collection to the subsequent detection of a genetically-related clinical isolate, defined by pairwise SNP distance ≤50 SNPs. Only food-clinical isolate pairs where the food isolate was collected before the clinical isolate were included in this analysis. Only food categories with at least 10

isolates were used in the analysis (i.e., isolates from sandwiches, raw fish and pasta were excluded due to limited sample sizes). For each clinical isolate, the minimum SNP distance was identified within each food category. The food category (or categories in the case the same SNP distance was observed between different categories linked to the same clinical isolate) with the smallest minimum SNP distance to that clinical isolate was kept, and food-clinical isolate pairs from other categories with higher SNP distances were excluded. All food-clinical isolate pairs within the kept category or categories were included in the analysis. A Cox proportional hazards model was used to estimate the time to cluster as a function of food category, and presence of *inlA* PMSC. The *Coxph* function from the *survival* R package was used, and the model assumptions were assessed using the *cox.zph* test. Multiple pairwise comparisons between food categories were evaluated using Tukey's post-hoc test for multiple contrasts using the *glht* function from the *multcomp* R package. Visualization of survival functions was conducted with Kaplan-Meier curves stratified by food source using the *survfit* and *ggsurvplot* functions from *survminer* R package.

7. Statistical analysis

All statistical analyses were performed in R version 4.4.0. Two-sided Fisher exact tests were used to evaluate associations (i) between CCs and clinical and nonclinical isolates, (ii) between CCs and food categories, as well as (iii) between presence of *inlA* PMSC and food categories. For each test, a 2 × 2 contingency table was constructed comparing the presence of a given CC in a specific category across other categories. Odds ratios (ORs) and corresponding 95% confidence intervals were obtained from Fisher exact tests, applying a continuity correction of 0.5 to all cells to allow for odds ratio calculation and ensure comparability across categories when necessary. Multiple testing correction was applied using Benjamini-Hochberg false discovery rate (FDR) method. Analyses were conducted using the *dplyr*, *purr*, *broom*, and *tidyr* R packages. All statistical tests were performed at a significance level of 0.05 and 95% confidence intervals were provided in the tables.

Appendix Results

To evaluate whether virulence and sanitizer tolerance markers were associated with food category, we conducted multinomial logistic regression analyses separately for selected CCs

(CC321, CC5, and CC9). For each CC, food category was modeled as the outcome variable, with *inlA* PMSC status and sanitizer tolerance gene presence as predictors. Overall, the results did not provide interpretable associations between these genetic markers and food categories (Appendix Table 5). Model estimates were characterized by very large standard errors and extreme coefficient values, indicating sparse data and limited sample sizes within specific CC-food category combinations. While some coefficients suggested potential differences in the distribution of PMSC or sanitizer tolerance across food categories, these patterns were not consistent and should not be overinterpreted. These findings indicate that, within the available dataset, there is insufficient statistical power to robustly link *inlA* PMSC presence or sanitizer tolerance genes to specific food categories within individual CCs.

References

1. Samut H, Mendez-Vallellanes DV, Hoyt H, Wirth SE, Mingle L, Sauders BD, et al. Retrospective analysis of historical Listeria monocytogenes clinical isolates, New York, USA, 2000–2021. Emerg Infect Dis. 2025;31:1883–92. PubMed https://doi.org/10.3201/eid3110.241876

2. Jagadeesan B, Baert L, Wiedmann M, Orsi RH. Comparative analysis of tools and approaches for source tracking Listeria monocytogenes in a food facility using whole-genome sequence data. Front Microbiol. 2019;10:947. PubMed https://doi.org/10.3389/fmicb.2019.00947

3. Stasiewicz MJ, Oliver HF, Wiedmann M, den Bakker HC. Whole-genome sequencing allows for improved identification of persistent *Listeria monocytogenes* in food-associated environments. Appl Environ Microbiol. 2015;81:6024–37. PubMed https://doi.org/10.1128/AEM.01049-15

4. Dickinson MC, Wirth SE, Baker D, Kidney AM, Mitchell KK, Nazarian EJ, et al. Implementation of a high-throughput whole genome sequencing approach with the goal of maximizing efficiency and cost effectiveness to improve public health. Microbiol Spectr. 2024;12:e0388523. PubMed https://doi.org/10.1128/spectrum.03885-23

5. Stevens EL, Carleton HA, Beal J, Tillman GE, Lindsey RL, Lauer AC, et al. Use of whole genome sequencing by the Federal Interagency Collaboration for genomics for food and feed safety in the United States. J Food Prot. 2022;85:755–72. PubMed https://doi.org/10.4315/JFP-21-437

6. Richardson LC, Bazaco MC, Parker CC, Dewey-Mattia D, Golden N, Jones K, et al. An updated scheme for categorizing foods implicated in foodborne disease outbreaks: a tri-agency

collaboration. Foodborne Pathog Dis. 2017;14:701–10. PubMed https://doi.org/10.1089/fpd.2017.2324

7. Souvorov A, Agarwala R, Lipman DJ. SKESA: strategic k-mer extension for scrupulous assemblies. Genome Biol. 2018;19:153. PubMed https://doi.org/10.1186/s13059-018-1540-z

8. Ragon M, Wirth T, Hollandt F, Lavenir R, Lecuit M, Le Monnier A, et al. A new perspective on *Listeria monocytogenes* evolution. PLoS Pathog. 2008;4:e1000146. PubMed https://doi.org/10.1371/journal.ppat.1000146

9. Liao J, Wiedmann M, Kovac J, Elkins CA. Genetic stability and evolution of the sigB allele, used for Listeria Sensu Stricto subtyping and phylogenetic inference. Appl Environ Microbiol. 2017;83:306–23. PubMed https://doi.org/10.1128/AEM.00306-17

10. Pettengill JB, Rand H, Wang SS, Kautter D, Pightling A, Wang Y. Transient and resident pathogens: intra-facility genetic diversity of Listeria monocytogenes and Salmonella from food production environments. PLoS One. 2022;17:e0268470. **PMID 36048885**

11. Pightling AW, Pettengill JB, Luo Y, Baugher JD, Rand H, Strain E. Interpreting whole-genome sequence analyses of foodborne bacteria for regulatory applications and outbreak investigations. Front Microbiol. 2018;9:1482. PubMed https://doi.org/10.3389/fmicb.2018.01482

12. Jackson KA, Gould LH, Hunter JC, Kucerova Z, Jackson B. Listeriosis outbreaks associated with soft cheeses, United States, 1998–2014. Emerg Infect Dis. 2018;24:1116–8. PubMed https://doi.org/10.3201/eid2406.171051

13. Chen Y, Gonzalez-Escalona N, Hammack TS, Allard MW, Strain EA, Brown EW. Core genome multilocus sequence typing for identification of globally distributed clonal groups and differentiation of outbreak strains of Listeria monocytogenes. Appl Environ Microbiol. 2016;82:6258–72. PubMed https://doi.org/10.1128/AEM.01532-16

14. Williams IT, Whitlock L, Wise ME. Acute enteric disease outbreaks. In: Rasmussen SA, Goodman RA, editors. The CDC field epidemiology manual. New York: Oxford University Press; 2019.

15. Shen W, Le S, Li Y, Hu F. SeqKit: a cross-platform and ultrafast toolkit for FASTA/Q file manipulation. PLoS One. 2016;11:e0163962. PubMed https://doi.org/10.1371/journal.pone.0163962

Appendix Table 1. Common *Listeria monocytogenes* CCs among food isolates. For each CC, the total number of food isolates (T) and the number of those cluster-linked (CL) with at least one clinical isolate (SNPs ≤50) are shown for each food category. For each food category, the number of the most predominant CC was shown in bold.

	Processed meat		Raw fish		Processed fish		Salad		Pasta	Raw meat		Processed dairy		Sandwiches		Produce		Raw dairy		Nuts	Number of isolates	
CC	T	CL	T	CL	T	CL	T	CL	T	T	CL	T	CL	T	CL	T	CL	T	CL	T	T	CL
CC1	3	1	2	0	4	0	0	0	0	0	0	2	0	1	0	0	0	**5**	0	0	17	1
CC2	1	0	2	0	0	0	1	0	0	0	0	1	0	0	0	0	0	0	0	0	5	0
CC3	3	0	0	0	5	0	0	0	**2**	0	0	0	0	1	0	0	0	0	0	0	11	0
CC4	0	0	0	0	0	0	0	0	0	0	0	0	0	0	0	0	0	1	0	0	1	0
CC5	13	1	1	0	7	1	**3**	1	0	**9**	1	2	1	1	0	0	0	0	0	0	**36**	5
CC6	4	4	0	0	0	0	0	0	0	0	0	**5**	3	0	0	0	0	4	0	0	13	7
CC7	1	1	1	0	0	0	0	0	0	1	0	1	0	0	0	**3**	0	0	0	0	7	1
CC8	0	0	**3**	0	1	1	0	0	0	1	1	0	0	0	0	0	0	1	0	0	6	2
CC9	**32**	0	0	0	4	0	0	0	0	0	0	0	0	0	0	0	0	0	0	0	**36**	0
CC11	2	2	0	0	1	1	0	0	0	1	0	1	0	0	0	0	0	2	0	0	7	3
ST13	1	0	0	0	0	0	0	0	0	0	0	0	0	0	0	0	0	0	0	0	1	0
CC14	0	0	0	0	0	0	0	0	0	0	0	1	0	0	0	1	0	1	0	0	3	0
CC18	0	0	0	0	1	0	0	0	0	0	0	0	0	0	0	0	0	0	0	0	1	0
CC19	1	1	0	0	0	0	0	0	0	0	0	0	0	0	0	0	0	0	0	0	1	1
CC20	0	0	0	0	1	0	0	0	0	0	0	0	0	0	0	0	0	0	0	0	1	0
CC29	0	0	0	0	0	0	0	0	0	0	0	0	0	0	0	0	0	2	0	0	2	0
CC31	0	0	0	0	0	0	0	0	0	0	0	2	0	0	0	0	0	0	0	0	2	0
CC37	0	0	0	0	1	0	0	0	0	0	0	2	0	0	0	1	0	1	0	0	5	0
CC59	0	0	1	0	1	0	0	0	0	1	0	0	0	0	0	0	0	0	0	0	3	0
CC88	2	0	1	0	5	0	0	0	1	0	0	0	0	0	0	0	0	0	0	0	9	0
CC89	0	0	0	0	0	0	0	0	0	0	0	0	0	0	0	0	0	1	0	0	1	0
CC101	0	0	0	0	1	0	0	0	0	0	0	0	0	0	0	0	0	0	0	0	1	0
CC121	2	0	2	0	**12**	3	0	0	0	1	0	0	0	0	0	0	0	1	0	0	18	3
CC155	3	3	2	1	2	1	1	0	0	1	1	1	0	2	2	0	0	0	0	0	12	8
CC191	0	0	0	0	0	0	0	0	0	1	0	0	0	0	0	0	0	0	0	0	1	0
CC193	0	0	0	0	1	0	0	0	0	0	0	0	0	0	0	0	0	0	0	0	1	0
CC199	3	0	**3**	0	5	0	0	0	0	0	0	0	0	0	0	2	0	0	0	**1**	14	0
CC203	0	0	0	0	1	0	0	0	0	0	0	0	0	0	0	0	0	0	0	0	1	0
CC204	0	0	0	0	1	1	1	1	0	0	0	0	0	0	0	0	0	0	0	0	2	2
ST213	0	0	0	0	0	0	0	0	0	1	0	0	0	0	0	0	0	0	0	0	1	0
CC224	0	0	0	0	2	0	1	0	0	0	0	0	0	0	0	0	0	0	0	0	3	0
CC226	0	0	2	0	3	0	0	0	0	0	0	0	0	0	0	0	0	1	0	0	6	0
CC288	2	0	0	0	1	0	0	0	0	2	0	0	0	0	0	0	0	0	0	0	5	0
CC321	16	14	2	1	10	10	1	1	0	0	0	1	1	**3**	3	0	0	0	0	0	33	30
CC369	0	0	0	0	0	0	0	0	0	0	0	0	0	0	0	**3**	0	0	0	0	3	0
ST376	0	0	0	0	0	0	0	0	0	0	0	0	0	0	0	0	0	1	0	0	1	0
CC379	0	0	0	0	0	0	0	0	0	0	0	1	0	0	0	0	0	3	0	0	4	0
CC388	0	0	0	0	0	0	0	0	0	0	0	0	0	0	0	0	0	2	0	0	2	0
CC389	0	0	0	0	0	0	0	0	0	0	0	0	0	0	0	0	0	4	0	0	4	0
CC392	1	0	0	0	0	0	0	0	0	0	0	0	0	0	0	0	0	1	0	0	2	0
CC403	0	0	0	0	0	0	0	0	0	0	0	0	0	0	0	0	0	1	0	0	1	0
CC426	0	0	0	0	1	0	0	0	0	0	0	0	0	0	0	0	0	0	0	0	1	0
CC466	0	0	0	0	0	0	0	0	0	0	0	0	0	0	0	1	0	0	0	0	1	0
CC489	1	0	0	0	0	0	0	0	0	0	0	0	0	0	0	0	0	0	0	0	1	0
CC554	0	0	0	0	1	1	0	0	0	0	0	0	0	0	0	0	0	2	1	0	3	2

CC	Processed meat		Raw fish		Processed fish		Salad		Pasta	Raw meat		Processed dairy		Sandwiches		Produce		Raw dairy		Nuts	Number of isolates	
	T	CL	T	CL	T	CL	T	CL	T	T	CL	T	CL	T	CL	T	CL	T	CL	T	T	CL
CC570	0	0	0	0	0	0	0	0	0	0	0	0	0	0	0	0	0	2	0	0	2	0
CC573	0	0	0	0	0	0	0	0	0	0	0	0	0	0	0	**3**	3	0	0	0	3	3
CC635	0	0	0	0	0	0	0	0	0	0	0	0	0	1	1	0	0	0	0	0	1	1
CC659	0	0	0	0	0	0	0	0	0	0	0	0	0	0	0	0	0	2	0	0	2	0
CC666	0	0	0	0	0	0	0	0	0	0	0	1	0	0	0	0	0	0	0	0	1	0
CC671	0	0	0	0	0	0	0	0	0	1	0	0	0	0	0	0	0	0	0	0	1	0
CC768	0	0	0	0	0	0	0	0	0	0	0	0	0	0	0	0	0	1	0	0	1	0
ST795	1	0	0	0	0	0	0	0	0	0	0	0	0	0	0	0	0	2	0	0	3	0
CC940	1	0	0	0	0	0	0	0	0	0	0	0	0	0	0	0	0	0	0	0	1	0
ST1046	0	0	0	0	0	0	0	0	0	1	0	0	0	0	0	0	0	0	0	0	1	0
CC1078	0	0	0	0	0	0	0	0	0	0	0	0	0	0	0	0	0	4	0	0	4	0
ST1649	0	0	0	0	0	0	0	0	0	0	0	0	0	0	0	0	0	1	0	0	1	0
ST2579	0	0	0	0	0	0	0	0	1	0	0	0	0	0	0	0	0	0	0	0	1	0
ST2628	0	0	0	0	0	0	0	0	0	0	0	0	0	0	0	1	0	0	0	0	1	0
Total	93	27	22	2	72	19	8	3	4	21	3	21	5	9	6	15	3	46	1	1	312	69

Appendix Table 2. Associations between *Listeria monocytogenes* selected clonal complexes (CC) with ≥10 food isolates (i.e., CC5, CC9, CC321, CC121, CC1, CC199, CC6, CC155, CC3) and reduced food categories (i.e., processed and raw food categories of meat, fish, and dairy) in New York State (2000–2021)*

Clonal Complex †	Food Category	Odds Ratio (95% CI) ‡	Adjusted p value (FDR)
CCs overrepresented among processed forms of corresponding food category compared to their raw forms			
CC1	meat	1.05 (0.05–21.72)	1
CC3	meat	1.05 (0.05–21.72)	1
CC3	fish	3.09 (0.16–59.76)	1
CC5	dairy	5.53 (0.23–130.34)	1
CC5	fish	1.35 (0.21–8.78)	1
CC6	meat	1.37 (0.07–27.18)	1
CC6	dairy	1.22 (0.23–6.39)	1
CC9	meat	15.74 (0.90–176.54)	0.077
CC9	fish	2.47 (0.12–49.07)	1
CC121	fish	1.4 (0.31–6.40)	1
CC155	dairy	3.00 (0.11–82.40)	1
CC199	meat	1.05 (0.05–21.72)	1
CC321	meat	5.98 (0.33–106.76)	0.775
CC321	dairy	3.00 (0.11–82.40)	1
CC321	fish	1.12 (0.24–5.20)	1
CCs overrepresented among raw forms of corresponding food category compared to their processed forms			
CC1	dairy	3.85 (0.61–25)	0.775
CC1	fish	2.38 (0.45–12.50)	1
CC5	meat	20 (4.17–100)	0.001
CC121	meat	4.35 (0.53–33.33)	1
CC121	dairy	3.57 (0.13–100)	1
CC155	fish	4.54 (0.69–33.33)	0.775
CC155	meat	3.12 (0.41–25)	1
CC199	fish	2.94 (0.66–14.28)	0.775

*Associations were assessed using two-sided Fisher exact test followed by false discovery rate (FDR) correction for multiple comparisons. Odds ratio and their 95% confidence intervals (CI) were reported.

†Some of the CC and food combinations were not shown here as CC9, CC199, and CC3 were not observed among any dairy isolates and CC6 was not observed among fish isolates.

‡A continuity correction of 0.5 was applied to all cells to allow for odds ratio calculation and ensure comparability across categories.

Appendix Table 3. Associations between food categories and presence of sanitizer tolerance genes (*bcrABC, Tn6188_qacH,* or *LGI1_LM5578_1862*) in food *Listeria monocytogenes* isolates from New York State (2000–2021)*

Food Category	Odds Ratio (95% CI) †	Adjusted p value (FDR)
Raw dairy	0.02 (0.00–0.30)	<0.001
Processed meat	1.28 (0.77–2.14)	0.430
Salad	3.68 (0.94–14.39)	0.134
Sandwiches	2.85 (0.80–10.15)	0.255
Processed dairy	0.27 (0.07–1.02)	0.104
Produce	0.22 (0.04–1.20)	0.121
Processed fish	3.00 (1.74–5.16)	0.001
Raw fish	1.64 (0.69–3.91)	0.403
Pasta	0.25 (0.01–4.61)	0.403
Raw meat	0.94 (0.36–2.42)	1.000
Nuts	0.75 (0.03–18.52)	1.000

*Odds ratios >1 indicate overrepresentation of the sanitizer tolerance genes in the corresponding food category. Associations were assessed using two-sided Fisher exact test followed by FDR correction for multiple comparisons.

†A continuity correction of 0.5 was applied to all cells to allow for odds ratio calculation and ensure comparability across categories.

Appendix Table 4. Pairwise comparisons of food categories based on Cox proportional hazards model estimates for time between a food isolate collection and subsequent detection of a genetically-related clinical isolate (SNPS ≤50)*

Food Category Pairs	Hazard Ratio (HR) (95% CI)	p value †
Processed fish - Processed dairy	1.66 (0.46–5.99)	0.902
Processed meat - Processed dairy	1.05 (0.32–3.39)	1.000
Produce - Processed dairy	9.72 (2.46–38.41)	<0.001
Raw dairy - Processed dairy	0.85 (0.22–3.36)	1.000
Raw meat - Processed dairy	0.31 (0.06–1.51)	0.296
Salad - Processed dairy	1.28 (0.27–5.96)	0.999
Processed meat - Processed fish	0.63 (0.28–1.43)	0.632
Produce - Processed fish	5.84 (1.92–17.72)	<0.001
Raw dairy - Processed fish	0.51 (0.16–1.64)	0.615
Raw meat - Processed fish	0.18 (0.04–0.76)	0.008
Salad - Processed fish	0.77 (0.21–2.86)	0.997
Produce - Processed meat	9.28 (3.32–25.94)	<0.001
Raw dairy - Processed meat	0.82 (0.29–2.31)	0.997
Raw meat - Processed meat	0.29 (0.08–1.1)	0.087
Salad - Processed meat	1.22 (0.35–4.23)	0.999
Raw dairy - Produce	0.09 (0.02–0.31)	<0.001
Raw meat - Produce	0.03 (0.01–0.14)	<0.001
Salad - Produce	0.13 (0.03–0.53)	<0.001
Raw meat - Raw dairy	0.36 (0.08–1.6)	0.394
Salad - Raw dairy	1.50 (0.35–6.33)	0.982
Salad - Raw meat	4.19 (0.8–21.93)	0.141

*An HR>1 means that the first food category in the pair has a higher linking probability to a subsequently detected clinical isolate (i.e., shorter time to link) compared to the second food category.
†p values were calculated using Tukey's post hoc test, which automatically controls for multiple pairwise comparisons.

Appendix Table 5. Multinomial regression results assessing associations between food category and genetic markers within selected clonal complexes (CC5, CC9, CC321)*

Clonal Complex	Food category comparison	Relative Risk Ratio	Lower 95% CI	Upper 95% CI
CC321	processed fish-processed dairy	8.04E+01	1.43E-314	Inf
CC321	processed fish-processed dairy	3.53E-01	4.72E-159	2.64E+157
CC321	processed meat-processed dairy	1.69E+02	0.00E+00	Inf
CC321	processed meat-processed dairy	3.08E-01	9.39E-180	1.01E+178
CC321	raw fish-processed dairy	6.57E-05	5.87E-110	7.35E+100
CC321	raw fish-processed dairy	1.23E+02	3.72E-51	4.10E+54
CC5	processed fish-processed dairy	3.19E-05	1.73E-05	5.89E-05
CC5	processed fish-processed dairy	1.74E+05	3.45E-285	8.77E+294
CC5	processed meat-processed dairy	6.84E+05	0.00E+00	Inf
CC5	processed meat-processed dairy	1.05E+05	2.09E-285	5.31E+294
CC5	raw fish-processed dairy	1.06E-01	4.54E-259	2.47E+256
CC5	raw fish-processed dairy	7.71E+12	8.39E-132	7.09E+156
CC5	raw meat-processed dairy	2.82E+06	0.00E+00	Inf
CC5	raw meat-processed dairy	1.52E+05	3.01E-285	7.67E+294
CC9	processed meat-processed fish	5.60E+01	2.47E+00	1.27E+03
CC9	processed meat-processed fish	1.07E-01	5.25E-03	2.19E+00

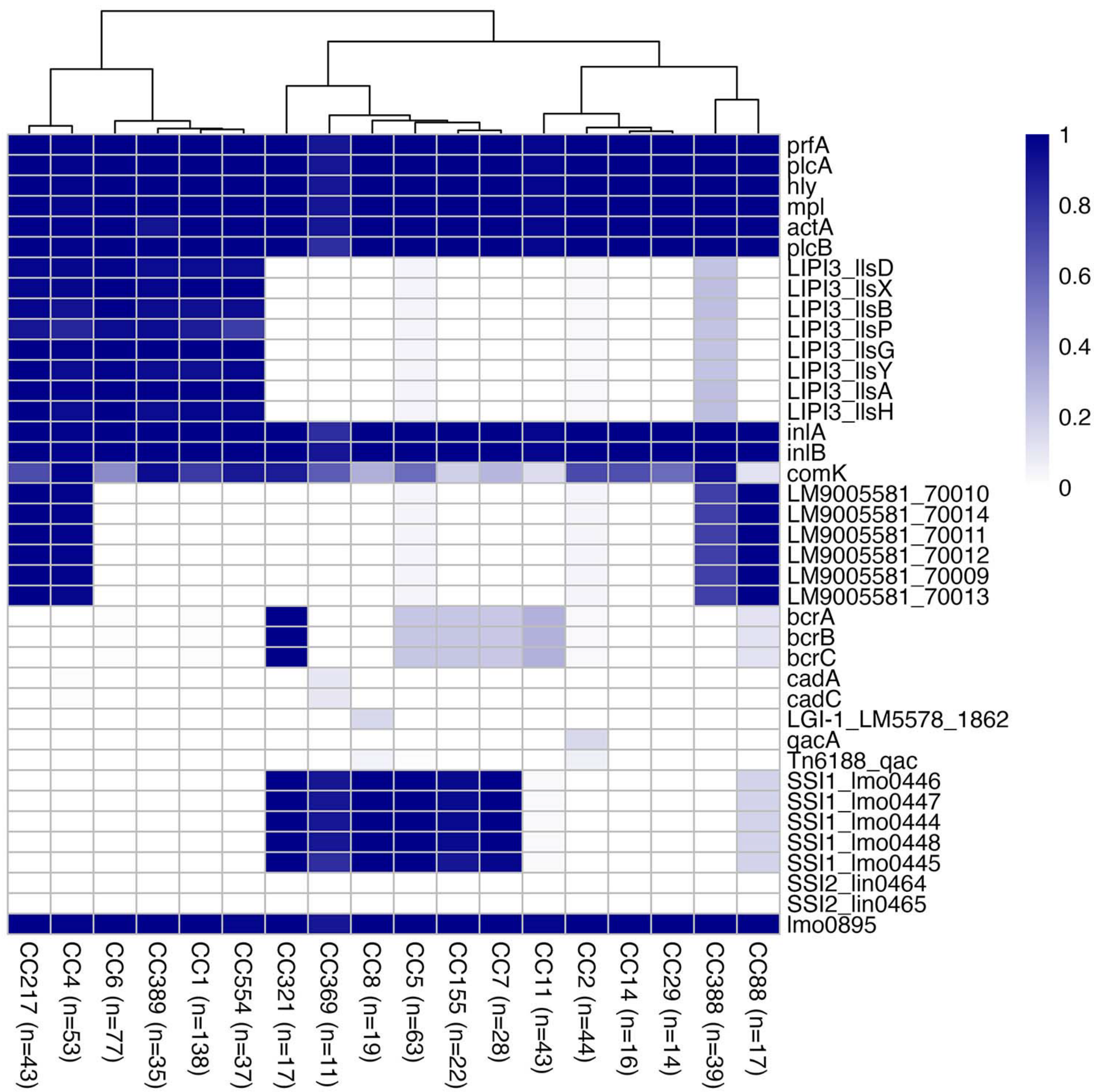

Appendix Figure 1. Heatmap showing the presence of virulence and tolerance-associated genes among clinical *Listeria monocytogenes* isolates. The isolates are grouped by CCs and only the CCs with more than 10 isolates are shown. Each row represents a gene and each column a CC, with color intensity corresponding to the proportion of isolates (dark blue represents high presence). The CCs are hierarchically clustered. The genes screened included virulence genes (*actA, hly, mpl, prfA, plcA, plcB, inlA, inlB,* LIPI-3 genes [*llsA, llsB, llsD, llsG, llsH, llsX, llsY, llsP*], LIPI-4 genes [*LM9005581_70009–70014*]), tolerance genes (*bcrABC, qacA, Tn6188_qac, cadA, cadC, LGI1_LM5578_1862*) and stress response genes SSI-1 (*lmo0444-lmo0448*), SSI-2 (*lin0464, lin0465*), and *comK.*

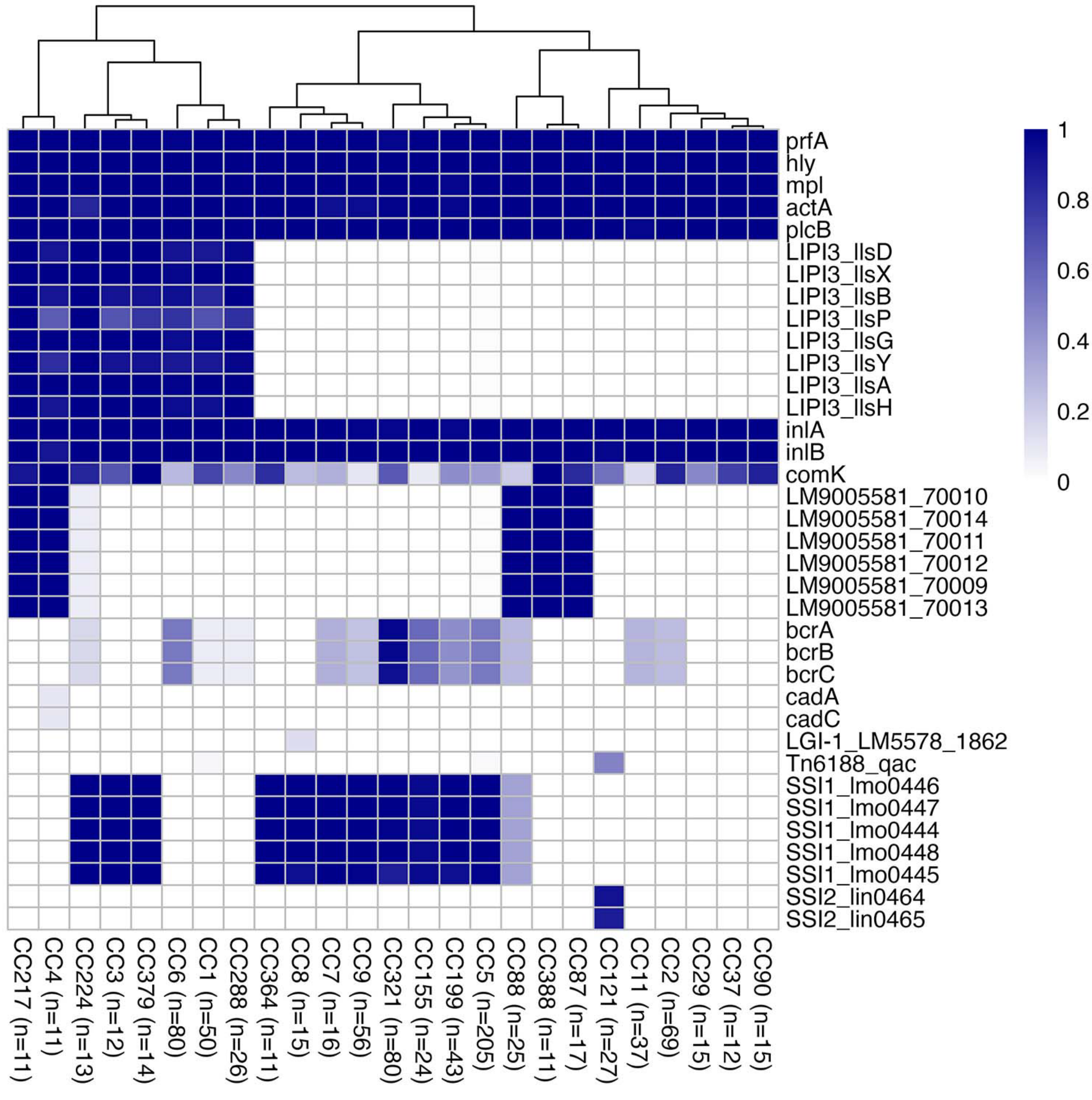

Appendix Figure 2. Heatmap showing the presence of virulence and tolerance-associated genes among nonclinical *Listeria monocytogenes* isolates. The isolates are grouped by CCs and only the CCs with more than 10 isolates are shown. Each row represents a gene and each column a CC, with color intensity corresponding to the proportion of isolates (dark blue represents high presence). The CCs are hierarchically clustered. The genes screened included virulence genes (*actA, hly, mpl, prfA, plcA, plcB, inlA, inlB,* LIPI-3 genes [*llsA, llsB, llsD, llsG, llsH, llsX, llsY, llsP*], LIPI-4 genes [*LM9005581_70009–70014*]), tolerance genes (*bcrABC, qacA, Tn6188_qac, cadA, cadC, LGI1_LM5578_1862*) and stress response genes SSI-1 (*lmo0444-lmo0448*), SSI-2 (*lin0464, lin0465*), and *comK.*
